# Preparation of Graft Poly(Arylene Ether Sulfone)s-Based Copolymer with Enhanced Phase-Separated Morphology as Proton Exchange Membranes via Atom Transfer Radical Polymerization

**DOI:** 10.3390/polym11081297

**Published:** 2019-08-02

**Authors:** Yang Zhao, Xue Li, Zhongyang Wang, Xiaofeng Xie, Wei Qian

**Affiliations:** 1 Institute of Nuclear and New Energy Technology, Tsinghua University, Beijing 100084, China; 2 Department of Energy, Environmental and Chemical Engineering, Washington University in Saint Louis, St Louis, MO 63130, USA; 3 Shanxi Research Institute for Clean Energy, Tsinghua University, Taiyuan 030032, China; 4 Foshan CleanEst Energy Technology Co. Ltd., Foshan 528225, China

**Keywords:** Poly(arylene ether sulfone)s, atom transfer radical polymerization (ATRP), proton exchange membrane (PEM), phase-separated morphology, fuel cell

## Abstract

Novel proton exchange membranes (PEMs) based on graft copoly(arylene ether sulfone)s with enhanced phase-separated morphology were prepared using atom transfer radical polymerization (ATRP). A series of PEMs with different graft lengths and sulfonation degrees were prepared. The phase-separated morphologies were confirmed by transmission electron microscopy. Among the membranes prepared and evaluated, PAESPS18S2 exhibited considerably high proton conductivity (0.151 S/cm, 85 °C), benefitting from the graft polymer architecture and phase-separated morphology. The membranes also possessed excellent thermal and chemical stabilities. Highly conductive and stable copoly(arylene ether sulfone)-based membranes would be promising candidates as polymer electrolytes for fuel cell applications.

## 1. Introduction

Proton exchange membrane (PEM), an essential component of proton exchange membrane fuel cells (PEMFCs), is considered to be an electrolyte for transporting protons from anode to cathode and as a separator to prevent the mixing of hydrogen and oxidant [1,2]. Currently, perfluorosulfonic acid membranes such as Nafion^®^ membranes are the most commonly used PEMs and have been regarded as the benchmarks for evaluating membrane performance because of their excellent chemical and electrochemical stability and outstanding proton conductivities [3]. However, inevitable problems of Nafion^®^ membranes such as high cost, remarkable fuel permeability, and restricted operating conditions (high temperatures and low relative humidity) have impeded the development of fuel cell technology [4]. Accordingly, extensive efforts have been devoted to developing alternative hydrocarbon-based PEMs to overcome the drawbacks of the perfluorosulfonic acid membranes [1,5]. Among these materials, sulfonated poly(arylene ether sulfone) is widely considered as a potential alternative due to its good thermal stability, strong mechanical properties, and excellent chemical stability. Meanwhile, the low fuel permeability, good proton conductivity, and low-cost features of sulfonated poly(arylene ether sulfone) (SPAES) make it an ideal substitute for Nafion^®^ membranes [6].

To date, studies of high-proton-conducting SPAES have shown that a promising way to enhance PEM performance and achieve hydration balance is to induce phase-separated morphology with hydrophilic and hydrophobic matrices. The phase separation feature of SPAES enhances the proton conductivities of PEM while maintaining reduced overall membrane hydration. Ding et al. [7,8,9], Holdcroft et al. [10], and Guiver et al. [11] investigated the structure–morphology–property relationships and came to the same conclusion that chemical structure and polymer morphology have a strong influence upon water sorption and proton transport properties. In addition, physical properties such as water uptake and dimensional swelling behavior also depend strongly on polymer morphology.

Given the evidence from existing research, constructing graft copolymers would be an effective way to introduce phase-separated morphology into PEMs. The architecture of these copolymers is comprised of a hydrophobic backbone with flexible hydrophilic ionic side chains, which is similar to state-of-the-art perfluorosulfonic acid PEMs (such as Nafion^®^ membranes). Their morphology can be controlled by varying the length and periodicity of side chains. Researchers have investigated various synthetic strategies to prepare graft copolymers employing PEMs [12,13,14]. Dramatic enhancement in proton conductivities under partially hydrated conditions was obtained for polymers with organized phase-separated morphology and well-connected nanochannels. Luo and coworkers [15] designed a series of polyphosphazene graft copolymer-based materials containing pendant alkyl sulfonated side chains. The membranes exhibited proton conductivity of 0.46–0.55 S/cm under fully hydrated conditions at 100 °C, which was 2.2–2.6 times higher than Nafion^®^ 117. Lafitte and coworkers [16] reported polysulfone-based copolymers containing sulfonated side chains. Compared to a random copolymer with two sulfonic acid groups on a polymer backbone, the copolymer with a short flexible side chain would have enhanced mobility and hydrophilicity over sulfonic acid groups. The property difference between a hydrophobic polymer backbone and hydrophilic sulfonic acid side chain leads to phase separation morphology of the PEM. 

In recent years, atom transfer radical polymerization (ATRP), as one of the prevalent living/controlled polymerizations, has been successfully used to prepare well-defined graft copolymers [17,18,19]. Generally, the ATRP reaction proceeds with organic halogens as the initiator and transition metal salts as the catalyst. During the polymerization process, the halogen atom is reversibly transferred and related to the dormant polymer chain [20]. Thus, this allows researchers to synthesize well-defined chains and functionalized polymers with controlled number average molecular weights and relatively narrow molecular weight distributions. Extensive and systematic research efforts have been devoted to synthesizing graft polymers with a sulfonated polystyrene side chain and the main chains of fluorinated polymers containing reactive C-Cl or C-Br bonds serving as macroinitiators for the ATRP graft reaction. Zhang et al. [21] reported the ATRP graft reaction of a series of poly(vinylidene fluoride)-*g*-sulfonated polystyrene (PVDF-*g*-SPS) graft copolymers. This graft copolymer morphology contributed positively to a high ion exchange capacity (IEC, 2.75 mmol/g) and resistance to excessive water swelling, which yielded notably higher proton conductivity. Holdcroft et al. [22,23,24,25] investigated how a polymer’s microstructure affected its morphology and properties under various environmental conditions from different perspectives. The results indicated that short ionic graft polymers could enhance the elastic forces in the matrix and inhibit excessive swelling. Dong and coworkers [26,27] reported using a PVDF macroinitiator-grafted glycidyl methacrylate or 4-styrene sulfonic acid and trimethoxysilyl propyl methacrylate to prepare PEMs based on ATRP. Similarly, nonfluorinated polymers bearing reactive benzyl bromide or benzyl chloride could also be used as the macroinitiators. Jin and coworkers [28] successfully synthesized poly(ether sulfone ether ketone ketone)-grafted poly(sulfopropyl methacrylate) (PESEKK-*g*-PSPMA) by ATRP. The synthesized PEM exhibited remarkable IEC and proton conductivity and preferable lower water uptake and methanol permeability than Nafion^®^ 117. It is quite facile to prepare PEMs from chloromethylated polysulfone by ATRP: However, the reagent in the chloromethylation reaction is harmful and carcinogenic to researchers. Studies related to polysulfone-based PEMs prepared via ATRP are still rare, and properties including proton conductivity and water uptake need to be further improved. 

In this paper, the preparation of graft copoly(arylene ether sulfone)s with enhanced phase-separated morphology via ATRP was studied. The basic polymers (poly(arylene ether sulfone)s) were obtained from polymerization of 4,4′-difluoro-diphenyl sulfone (DFDPS) and 2,2′-bis(4-hydroxy phenyl) propane (4M-BPA). Then, the macroinitiator (bromoethylated poly(arylene ether sulfone)) was from the product of the reaction of N-bromosuccinimide (NBS) and the basic polymer, which avoided the use of carcinogenic reagents. Subsequently, ATRP was used to synthesize a series of graft copolymers from styrene and the macroinitiator, after which acetyl sulfate was used to prepare the sulfonated graft copolymers with various degrees of functionalization. The ion exchange capacity, water uptake, and proton conductivity of the PEMs were measured and compared to reveal the structure-property relationships in this class of PEMs.

## 2. Experiment

### 2.1. Materials

DFDPS was purchased from J&K Scientific chemical company and recrystallized from toluene before use. In addition, 4M-BPA and benzoyl peroxide (BPO) were purchased from J&K Scientific chemical company (Beijing, China). NBS, copper bromide (CuBr), 2,2′-bipyridyl (Bpy), and styrene were obtained from Shanghai Aladdin Chemical Reagents Company (Shanghai, China). Potassium carbonate (K_2_CO_3_), toluene, dimethyl sulfoxide (DMSO), 1-methyl-2-pyrrolidinone (NMP), dichloroethane (DCE), n-hexane, methanol, concentrated sulfuric acid (H_2_SO_4_), and hydrochloric acid (HCl) were purchased from Beijing Reagent Company (Beijing, China) and used as received without further purification. Deionized water made in the laboratory was used for all the experiments.

### 2.2. Synthesis of Poly(Arylene Ether Sulfone) (PAES)

A typical procedure was as follows. A 100-mL three-neck flask equipped with a mechanical stirrer, Dean-Stark trap, and an argon gas inlet was filled with DFDPS (6.356 g, 25 mmol), 4M-BPA (7.110 g, 25 mmol), NMP (43 mL), and toluene (28 mL) in sequence. The mixture was then stirred for 2 h while argon was purged at room temperature to obtain a homogeneous solution without oxygen. K_2_CO_3_ (4.364 g, 31.25 mmol) was added into the solution and continuously stirred for 1 h at room temperature before the temperature was increased to 140 °C and held for 3 h. After dehydration and the removal of toluene for several hours, the reaction temperature was then increased to about 170 °C and held for 20 h. The reaction mixture was cooled down to 40 °C and diluted with 45 mL of NMP, followed by a large excess of deionized water being poured in while the mixture was stirred vigorously. The resulting fibrous copolymer (PAES) was washed thoroughly with water and methanol alternately and was dried in a vacuum oven at 100 °C for 24 h. 

### 2.3. Synthesis of Bromomethylated Poly(Arylene Ether Sulfone) (BrPAES)

A typical procedure for preparing the bromomethylated copolymer is described as follows. The PAES (1.0 g, containing 8 mmol -CH_3_) was dissolved in 60 mL of DCE and was added into a 100-mL three-necked round-bottomed flask equipped with a magnetic stir bar, an argon inlet/outlet, and a condenser. The mixture was stirred for 2 h while purging argon at room temperature to get the homogeneous solution without oxygen. Then the bromination agent NBS (80.0 mg, 0.450 mmol) and initiator BPO (5.20 mg, 0.021 mmol) were added into the flask. Then the solution was heated and kept at reflux for 6 h. Upon cooling to room temperature, the solution was poured into 500 mL of methanol while being agitated vigorously. Finally, the product was dried at 70 °C in the vacuum oven. 

### 2.4. Preparation of Poly(Arylene Ether Sulfone)-Graft-Polystyrene Copolymers (PAESPSx)

ATRP of styrene was carried out by modifying the procedure reported in the literature [29,30]. After BrPAES (0.2 g) and a certain amount of styrene (1.2 mL, 1.5 mL, 1.8 mL) were dissolved in 13 mL of NMP in a 25-mL Schlenk flask, the polymer solution was degassed by three freeze–pump–thaw cycles. Then Bpy (58.6 mg, 0.375 mmol) was dissolved in 1 mL of NMP and transferred into the polymer mixture with the protection of inert gas. Separately, CuBr (21.6 mg, 0.150 mmol) was added into the polymer solution. The polymerization reaction was carried out at 110 °C for 12 h under an argon blanket. Polymerization was stopped by opening the flask and exposing the catalyst to air. The product was isolated by precipitation in methanol and the corresponding copolymers and was named PAESPS*x,* where *x* corresponds to the amount of styrene used in the graft reaction.

### 2.5. Sulfonation of Poly(Arylene Ether Sulfone)-Graft-Polystyrene Copolymers (PAESPSxSy) 

Sulfonation of PAESPS*x* was carried out according to the procedure described by Kim et al. [31], with a slight modification. In a 50-mL Schlenk flask, 0.5 g of the PAESPS*x* copolymer was dissolved into 25 mL of DCE. The flask was connected to the Schlenk line to degas the mixture via three freeze-evacuate-thaw cycles with argon. The sulfonating reagent was acetyl sulfate, which was prepared by a reaction of acetic anhydride and concentrated sulfuric acid at 0 °C in DCE. Then the freshly prepared acetyl sulfate was added dropwise to the polymer solution at 50 °C. After the desired reaction time, 5 mL of methanol was slowly added to terminate the reaction. The solvent in the reaction mixture was evaporated under reduced pressure, and the reaction product was recovered by precipitation in ice-cold deionized water. The sulfonated copolymer (PAESPS*x*S*y,* where *y* stands for the sulfonation time) was filtered and washed with deionized water until a neutral pH of wash water was obtained. The final product was dried in a vacuum oven at 80 °C for 24 h.

### 2.6. Preparation of Membranes

The membranes were prepared by casting a 1 wt % solution of sulfonated PAESPS*x* copolymers in NMP onto a clean glass plate and evaporating the solvent by drying the film at 65 °C for 12 h. The films were then dried at 100 °C for 8 h in a vacuum oven. After being cooled down to room temperature, the glass plate with cast membranes was soaked in deionized water, and the membranes were peeled off. The thicknesses of the membranes were 20–30 μm. The resulting film was treated by immersing it into 0.5 mol/L of H_2_SO_4_ for 24 h, washing it with water thoroughly, and drying it at room temperature.

### 2.7. Characterizations

FT-IR spectra were obtained with a Shimadzu-FTIR-8400 Fourier Transform Infrared Spectrophotometer (Tsinghua University, Beijing, China) to confirm the synthesis of the copolymers.

^1^H spectra of the copolymers were measured on a Bruker AVANCE NMR spectrometer (Tsinghua University, Beijing, China) using CDCl_3_ as a solvent and tetramethylsilane (TMS) as an internal reference.

Thermogravimetric analysis (TGA) and derivative thermogravimetric analysis (DTA) were performed on a PerkinElmer 7 series thermal analysis system (Tsinghua University, Beijing, China) at a heating rate of 10 °C/min under an N_2_ atmosphere [32].

IEC, water uptake, and in-plane proton conductivity of the membranes were determined by conducting the established procedure reported in the literature [33].

TEM observations were conducted using the lead stain method to displace the hydrogen in sulfonic acid groups with lead ions by immersing the samples into 0.5 mol/L of lead acetate aqueous solution for 24 h and then rinsing them with deionized water. The membrane samples were embedded in epoxy resin, sectioned to 80–100 nm thickness with a Leica Microtome, and tested on copper grids with a JEOL JEM-2100F transmission electron microscope (Tsinghua University, Beijing, China).

## 3. Results and Discussions

### 3.1. Synthesis of the Copolymers

**Scheme 1:**
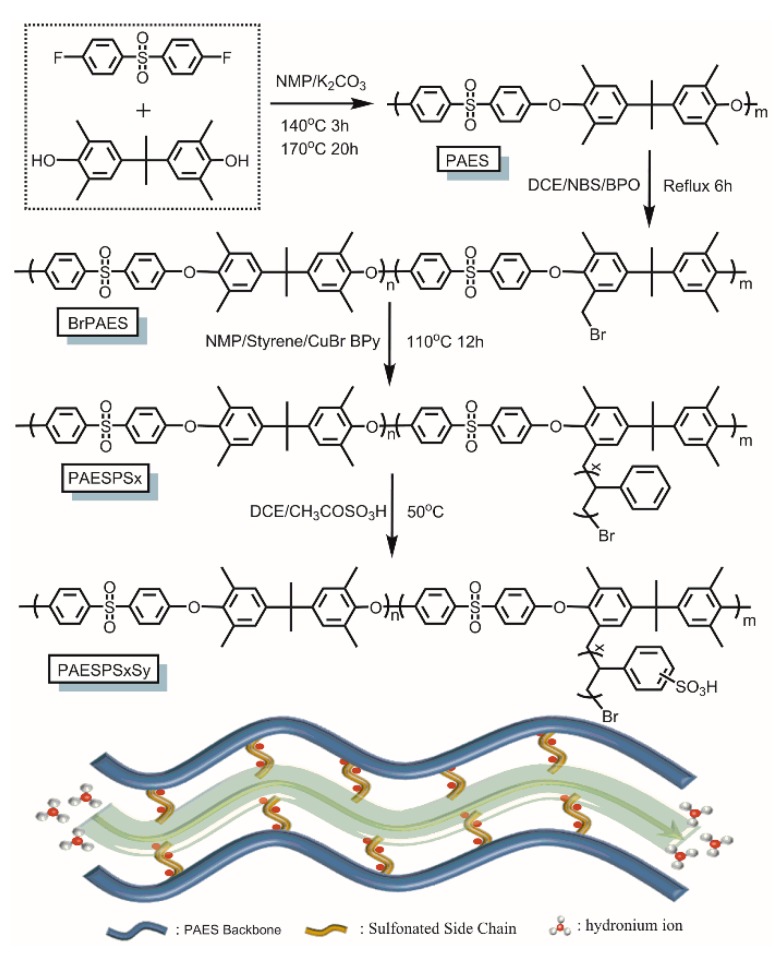
Synthesis scheme of poly(arylene ether sulfone) (PAES) membranes and an illustration of proton transport pathways through the membrane.

Scheme 1 shows the routine of the approach used to prepare the sulfonated copoly(arylene ether sulfone)s. It begins with the nucleophilic substitution reaction between DFDPS and 4M-BPA. The obtained PAES possessed a high molecular weight, making the decrease of the membrane thickness possible, and the benzyl methyl groups could serve as the reactive sites for the following bromination reaction. Subsequently, the macroinitiator (BrPAES) could be obtained from the bromination reaction using NBS as the bromination reagent and BPO as the initiator. Next, ATRP was used to synthesize graft copolymers from styrene and the macroinitiator with different side-chain lengths. In this graft system, monomer units were grown as side chains from multiple initiating sites along the backbone. Finally, the graft copolymers were sulfonated by acetyl sulfate. It has been reported that the sulfonation of styrene-containing polymers with acetyl sulfate resulted in selective sulfonation at the para-position of the aromatic ring in the styrene unit [34]. The number of sulfonic acid groups could be controlled by the ratio of sulfonated reagents and the reaction time. 

### 3.2. Structural Characterization of the Copolymers

**Figure 1:**
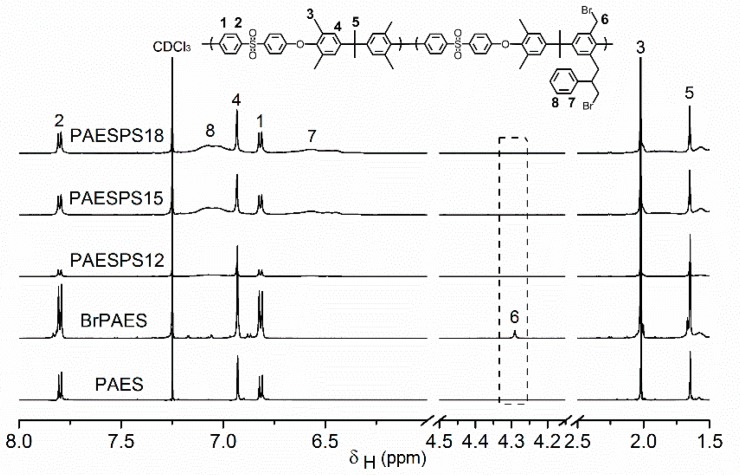
^1^H NMR spectra of PAES-based copolymers.

^1^H NMR spectra of PAES, BrPAES, and PAESPS*x* were collected (Figure 1) to confirm the successful synthesis of copolymers according to the position of several specific protons. The peak at about 2.17 ppm was assigned to the methyl group of PAES. Compared to the spectra of PAES, the new signal (peak 6) at around 4.43 ppm in the spectra of BrPAES was assigned to the protons in bromomethyl groups. The degree of functionalization of the brominated (*D_Br_*) copolymers was calculated by the following Equation (1),

(1) $$D_{\mathrm{Br}}\;=\;\frac{3A(6)}{3A\left(6\right)\;+\;2A(3)},$$

where *A(6)* is the peak area of the protons in bromomethyl groups, and *A(3)* is assigned to the peak area of methyl protons. The value of *D_Br_* was about 3.67%. After the ATRP reaction, this peak disappeared. The ^1^H NMR spectra also provided evidence for the growth of polystyrene from the PAES backbone: The spectra of PAESPS12, PAESPS15, and PAESPS18 showed additional peaks at ~6.55 ppm (peaks 7, aryl, 2H) and ~7.14 ppm (peaks 8, aryl, 3H). Quantitative analysis of the number of styrene units was obtained by the ratio of integrated signals from the aromatic styrenic protons (peaks 7 and 8) and the methylene protons of the mainchain (peak 5), as follows:

(2) $$n=\frac{3\left[A\left(7\right)+A\left(8\right)\right]}{10A\left(5\right)}\times \frac{1}{D_{B_{r}}},$$

where *A(7)* and *A(8)* are the peak areas of aromatic styrene protons, and *A(5)* is assigned to the peak of methyl protons. During the ATRP graft reaction, various amounts of styrene were added to control the graft length (average styrene repeating units per polystyrene graft) in the graft copolymer. The corresponding data about graft length estimated from the spectra is summarized in Table 1. With the increase in feed ratio, the number of styrenes increased steadily. The reason for the above results was mainly attributed to the catalytic activity of CuBr/BPy. Due to the poor solubility in (CD_3_)_2_SO, the sulfonated polymers could not be characterized by NMR. Accordingly, we chose the IEC to calculate the sulfonation degree. It was obvious that the sulfonation degree of the copolymer was quite dependent on the sulfonation reaction time for the same graft length of polystyrene.

**Figure 2:**
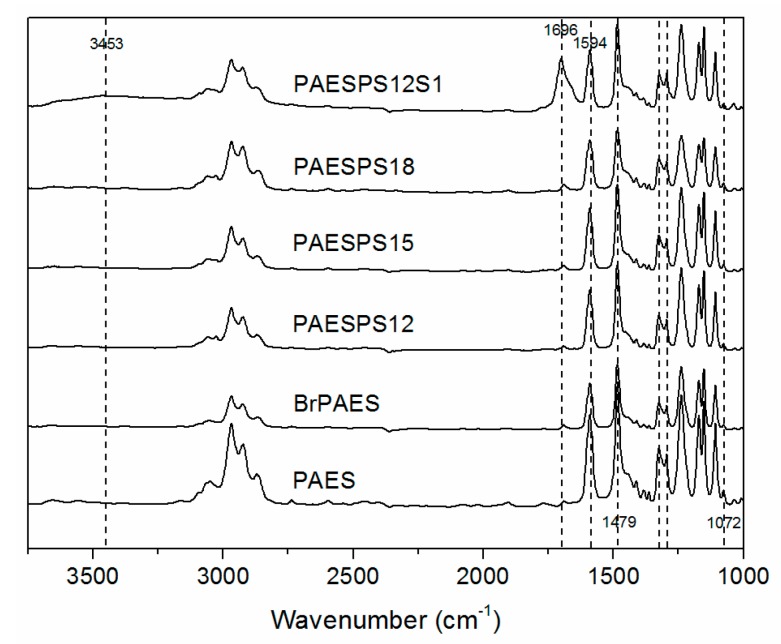
FT-IR spectra of PAES-based copolymers.

Figure 2 shows the FTIR spectra of PAES, BrPAES, PAESPS*x*, and PAESPS12S1. The absorption bands observed around 1594 cm^−1^ and 1479 cm^−1^ were assigned to the characteristic absorption of benzene rings in the polymers. When the substituent groups were attached to the aromatic rings, the bands at 1696 cm^−1^, assigned to the C=C stretching, became more obvious. The bands at 1326 and 1295 cm^−1^ were assigned to the asymmetric O=S=O stretching in the sulfone groups, and the band at 1072 cm^−1^ was assigned to the symmetric O=S=O stretching. In addition, a broad O-H stretching band appeared around 3400 cm^−1^, indicating the presence of water attracted by the hygroscopic sulfonate groups. Since those peaks were all attributed to the presence of sulfonic acid groups, the successful sulfonation of the hydrophilic oligomer was confirmed.

### 3.3. Characterization of Morphology

**Figure 3:**
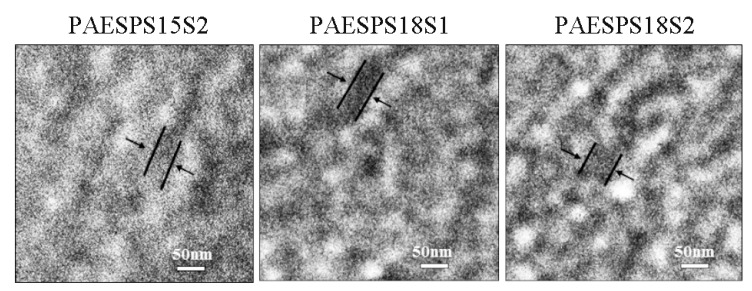
TEM images of the PAESPS15S2, PAESPS18S1, and PAESPS18S2 membranes.

The distinct morphologies were obtained by TEM from cross-sections of the membranes. The samples were prepared by soaking them in 0.5 mol/L lead acetate solution for 24 h to stain the ionic domains and then embedding them into epoxy resin followed by a microtome into 80–100 nm thicknesses. In order to cope with their working conditions, the membranes used for characterization were in a hydrous state rather than an anhydrous state. In Figure 3, the dark and bright regions correspond to the hydrophilic clusters of sulfonic acid groups and the hydrophobic clusters of the polymer backbone, respectively. The cross-sectional images of membranes exhibited clear hydrophilic/hydrophobic phase separation morphology. Membrane PAESPS15S2 and membrane PAESPS18S1, which had similar IECs (1.50 and 1.47 mmol/g, respectively), had similarly sized hydrophilic clusters, approximately 37 nm. With an increase in the number of sulfonic acid groups, the size and the connectivity of the hydrophilic clusters became obvious, increasing to about 45 nm. This well-defined phase-separated structure likely originated from the graft copolymer structure, which facilitated phase separation between hydrophilic and hydrophobic aggregates to form nanochannels and was expected to provide a pathway for efficient proton transporting. 

### 3.4. Ion Exchange Capacity

**Figure 4:**
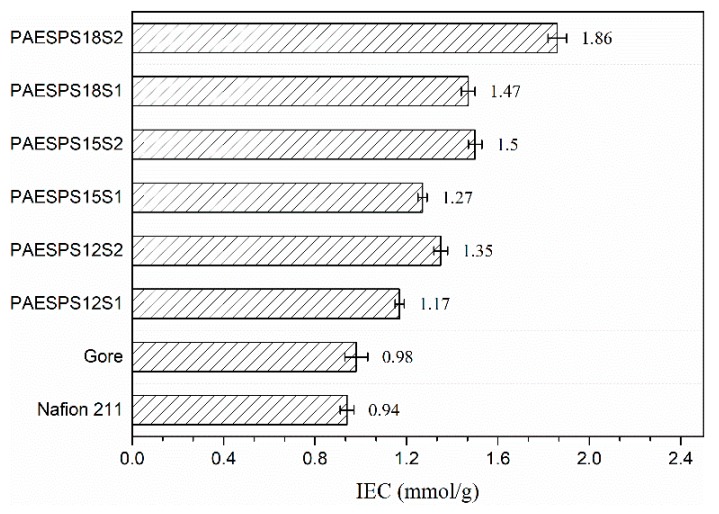
IECs of the PAES-based membranes, Gore, and the Nafion^®^ 211 membrane.

The IECs of the commercial membranes and the PAES membranes are shown in Figure 4. The degree of sulfonation was controlled by the sulfonation reaction time under the same amount of sulfonating agent. The sulfonation reaction time and the graft length of the polystyrene side chain played a role in the IEC. For example, for PAESPS12, when the sulfonation reaction time extended from 1 h to 2 h, the IEC increased from 1.17 mmol/g to 1.35 mmol/g. For the same sulfonation reaction time (1 h), the IECs of the PAESPS12S1, PAESPS15S1, and PAESPS18S1 copolymers exhibited an increasing trend (1.17 mmol/g, 1.27 mmol/g, and 1.47 mmol/g, respectively). Essentially, this was because the copolymers with a longer polystyrene side chain provided more reaction sites for sulfonation reactions under the same sulfonate conditions. In addition, it was notable that the PAES-based membranes exhibited higher IECs than those of Gore and Nafion^®^ 211. This was mainly attributed to the use of acetyl sulfate, which is a relatively soft sulfonating agent that could avoid the occurrence of side reactions [35].

### 3.5. Water Uptake

**Figure 5:**
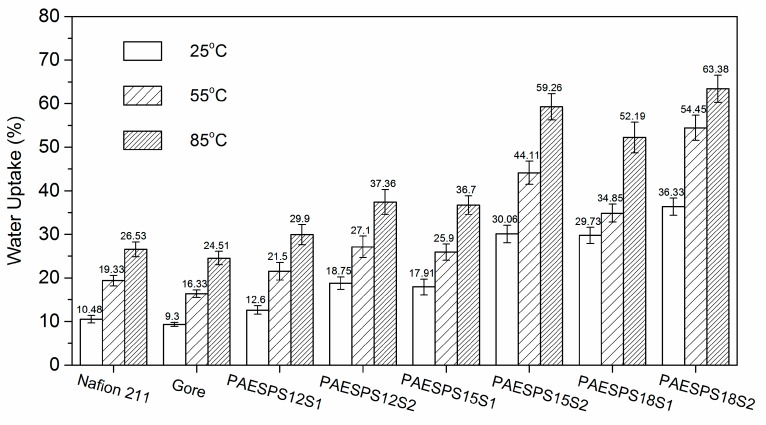
Water uptake of PAES-based membranes, Gore, and the Nafion^®^ 211 membrane at different temperatures.

The water uptake of the membrane is a very important property that directly affects proton transport through the membrane. Water can act as the carrier and medium for a proton as it moves through the membrane. However, excess water uptake can lead to a decrease in mechanical strength and dimensional distortion for fuel cell assembly. The temperature dependence of the water uptake for the commercial membranes and the PAES-based membranes is shown in Figure 5. As the IEC increased, the water uptake increased due to the increase in the hydrophilic segments in the copolymer. As the free volume increased with temperature for all copolymers, the water uptake also increased. All the PAES-based membranes held relatively higher IEC values than those of Gore and Nafion^®^ 211 under the same measured temperature. Nevertheless, the water uptake of all membranes remained below 70%, even at high temperature of 85 °C, in this study.

### 3.6. Proton Conductivity

**Figure 6:**
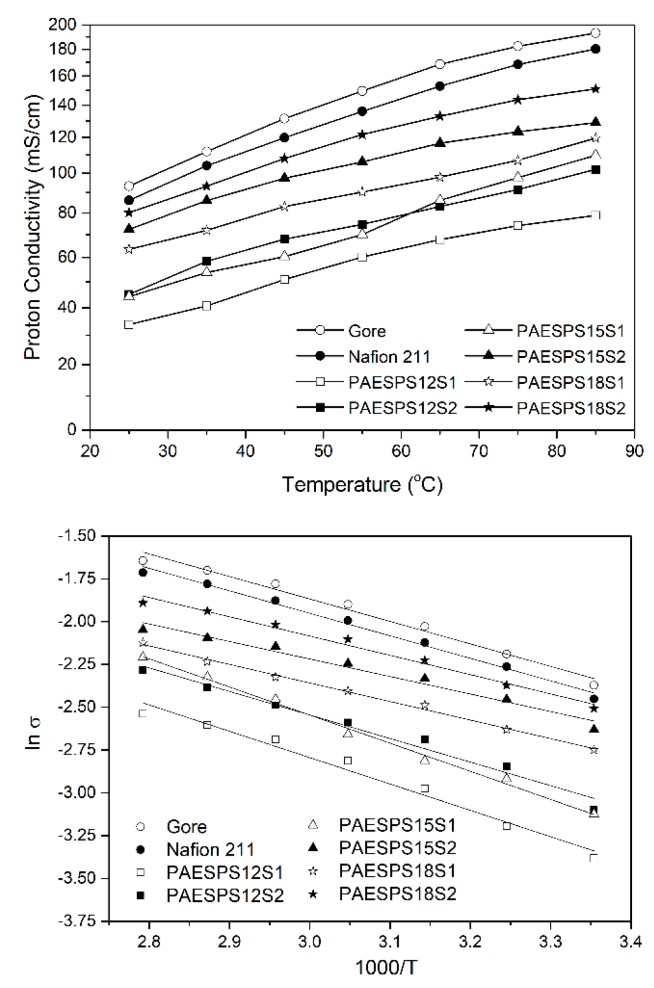
Proton conductivities of PAES-based membranes, Gore, and the Nafion^®^ 211 membrane at different temperatures.

Figure 6 shows the effect of temperature on membrane conductivity. The proton conductivities of the Nafion^®^ 211 and Gore membranes were also measured under the same experimental conditions and served as the benchmark. Admittedly, perfluorosulfonic acid membranes (Nafion^®^ 211 and Gore) possessed outstanding proton conductivity under low IECs due to their enhanced phase-separated morphology and strong acidic groups (pKa ≈ −6) [36]. Relatively, the proton conductivity of the Gore membrane was slightly higher than that of Nafion^®^ 211 at all temperatures due to the decreasing membrane thickness. Compared to commercial membranes, PAESPS*x*S*y* membranes showed relatively low conductivities, though with relatively high IECs. This was mainly due to the lower pKa of benzenesulfonic acid (pKa ≈ −1) in comparison with the perfluorosulfonic acid membranes. When the IEC of PAESPS18S2 elevated to 1.86 mmol/g, the proton conductivity increased to 0.08 S/cm at 25 °C, which was comparable to the Nafion^®^ 211 and Gore membranes. This was precisely due to the well-defined phase-separated morphologies and nanochannel pathways in the membrane. 

Remarkably, PAESPS*x*S*y* membranes with long side chains more easily obtained high conductivities because more reaction sites were available for the sulfonation reaction. For instance, with the same amount of sulfonation reaction time, membrane PAESPS18S1 had higher conductivity than membrane PAESPS15S1, and PAESPS15S1 was higher than PAESPS12S1 in all temperature ranges of measurement. The same changing rule was also applicable to membrane PAESPS12S2, membrane PAESPS15S2, and membrane PAESPS18S2. The regulation of the proton conductivity could be controlled by the graft length of the side chain within a certain sulfonation reaction time. Meanwhile, the sulfonation reaction time also had great influence on the proton conductivity of copolymers with the same graft length of side chain. This result corresponded to the trend of IECs mentioned above. 

Figure 6 also displays Arrhenius plots of the conductivities of the membranes measured at different temperatures. The activation energies calculated from the slope of the Gore and Nafion^®^ membranes were 10.91 and 10.93 kJ·mol^−1^, respectively. The similar values revealed that they had an identical matrix of materials. The activation energies of the membranes PAESPS12S1, PAESPS12S2, and PAESPS15S1 were 12.79, 11.43, and 13.64 kJ·mol^−1^, respectively. These membranes showed a little higher activation energy than those of the Gore and Nafion^®^ 211 membranes. In other words, these membranes had relatively low proton conduct abilities even though they held higher IECs than the perfluorosulfonic acid membranes. The activation energies of membrane PAESPS15S2, membrane PAESPS18S1, and membrane PAESPS18S2 were 8.50, 9.01, and 9.36 kJ·mol^−1^, respectively, all of which were lower than those of the Gore and Nafion^®^ 211 membranes, indicating that the protons would stride across lower energy barriers. 

### 3.7. Thermal Stability

**Figure 7:**
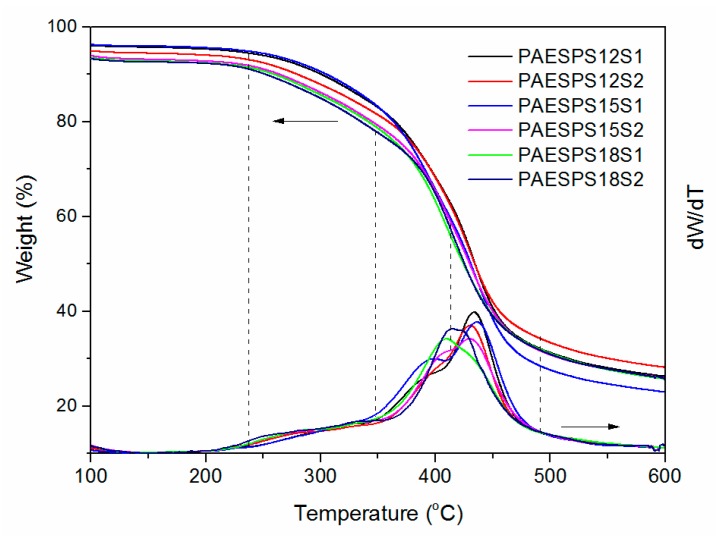
TGA and DTG curves of PAES-based membranes.

Figure 7 shows the TGA curves of PAESPS*x*S*y* in sulfonic acid form, which appeared to have three distinct degradation steps. The initial degradation step observed at around 240–350 °C was likely associated with the thermal degradation of the sulfonic acid groups. The second degradation step observed at around 350–400 °C was likely associated with the thermal degradation of the aliphatic backbone in the side chain. In addition, the third step starting at about 420 °C corresponded to the decomposition of aromatic backbone. The degradation temperature range was similar for all membranes because the IEC was essentially the same and the backbone was not changed. All the membranes had excellent thermal stabilities and could adequately meet the temperature requirements both for membrane electrode assembly (MEA) processing and for PEMFC devices.

## 4. Conclusions

Polystyrenes were successfully grafted onto poly(arylene ether sulfone) backbone by the ATRP reaction. Membranes with different graft lengths of side chains and degrees of sulfonation were designed and prepared systematically. Under the same sulfonation time, higher IECs and proton conductivities were acquired for copolymers with longer side chains. Similarly, both the IEC and proton conductivity had an obviously positive correlation with sulfonation time for copolymers with the same side chain length. In addition, a morphological study using TEM indicated that enhanced phase-separated morphology and interconnected ionic channels were formed in these PAES membranes. As expected, the proton conductivity of these membranes depended on IEC and the level of the connectivity of the ionic phases resulting from the morphology. Moreover, these PAES-based membranes also showed excellent thermal and chemical stabilities. Overall, the results of the present study demonstrated useful implications for novel proton exchange membranes. While solubility of the sulfonated copolymer can only be achieved at high temperatures, the chemical structure of the copolymer should be optimized in future research.

## Figures and Tables

**Table 1:** The graft and functionalization parameters of the different copolymers.

Copolymers ^a^	Feed Ratio ^b^	Graft Degree ^c^	Graft Length ^d^	Sulfonation Time (h)	Sulfonation Degree ^e^
PAESPS12S1	0.2:1.2	21.74%	15.82	1	41.80%
PAESPS12S2				2	49.01%
PAESPS15S1	0.2:1.5	32.93%	20.51	1	38.68%
PAESPS15S2				2	46.64%
PAESPS18S1	0.2:1.8	43.95%	26.14	1	39.50%
PAESPS18S2				2	51.81%

^a^ The copolymers were named in the form of PAESPS*x*S*y*. The *x* is the volume of styrene in the feed ratio, and the *y* represents the sulfonation time. ^b^ The feed ratio is mass (BrPAES, g)/volume (styrene, ml). ^c^ The graft degree is calculated from the net mass increment of copolymers before and after the atom transfer radical polymerization (ATRP) reaction. ^d^ The graft length is calculated from the results of ^1^H NMR. ^e^ The sulfonation degree is calculated from the results of the ion exchange capacity (IEC). The value is equal to mols of sulfonated styrene/mols of total styrene.
